# Center Bias Does Not Account for the Advantage of Meaning Over Salience in Attentional Guidance During Scene Viewing

**DOI:** 10.3389/fpsyg.2020.01877

**Published:** 2020-07-28

**Authors:** Candace E. Peacock, Taylor R. Hayes, John M. Henderson

**Affiliations:** ^1^Center for Mind and Brain, University of California, Davis, Davis, CA, United States; ^2^Department of Psychology, University of California, Davis, Davis, CA, United States

**Keywords:** attention, scene perception, eye movements, meaning, image salience

## Abstract

Studies assessing the relationship between high-level meaning and low-level image salience on real-world attention have shown that meaning better predicts eye movements than image salience. However, it is not yet clear whether the advantage of meaning over salience is a general phenomenon or whether it is related to center bias: the tendency for viewers to fixate scene centers. Previous meaning mapping studies have shown meaning predicts eye movements beyond center bias whereas saliency does not. However, these past findings were correlational or *post hoc* in nature. Therefore, to causally test whether meaning predicts eye movements beyond center bias, we used an established paradigm to reduce center bias in free viewing: moving the initial fixation position away from the center and delaying the first saccade. We compared the ability of meaning maps and image salience maps to account for the spatial distribution of fixations with reduced center bias. We found that meaning continued to explain both overall and early attention significantly better than image salience even when center bias was reduced by manipulation. In addition, although both meaning and image salience capture scene-specific information, image salience is driven by significantly greater scene-independent center bias in viewing than meaning. In total, the present findings indicate that the strong association of attention with meaning is not due to center bias.

## Introduction

**FIGURE 1:**
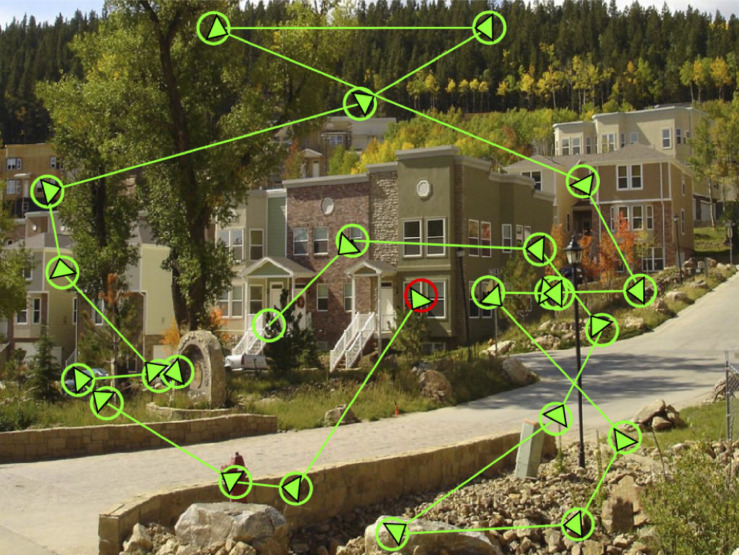
Participant scan path in a real-world scene. The red circle represents the first fixation and the green circles represent subsequent fixations. Arrows represent the trajectory of eye movements to the next landing point.

As we explore the visual world, our eyes move intelligently to prioritize the most important scene regions for fixation (Figure 1). Exactly how one scene region is prioritized over another remains an open question. Previous research using image saliency models has focused on the role of bottom-up, stimulus-driven processing on real-world attention allocation (Koch and Ullman, 1987; Itti et al., 1998; Itti and Koch, 2001; Harel et al., 2006; Borji et al., 2013, 2014). It is also well established that top-down factors related to viewing task can influence attentional selection processes (Buswell, 1935; Yarbus, 1967; Henderson and Hollingworth, 1999; Hayhoe et al., 2003; Hayhoe and Ballard, 2005; Navalpakkam and Itti, 2005; Henderson, 2007, 2017; Tatler et al., 2011; Rothkopf et al., 2016). What has been less clear is how the intrinsic semantic properties of a scene might influence eye movements and attention during scene viewing.

To investigate this issue, Henderson and Hayes (2017) introduced the concept of meaning maps. In the same way that saliency maps represent the spatial distribution of contrasts in image features, meaning maps capture the spatial distribution of semantic information in real-world scenes. In studies directly comparing meaning maps and saliency maps, meaning has been found to be a significantly better predictor of visual attention than image salience. This advantage for meaning over salience was observed across viewing tasks such as aesthetic judgment and memorization (Henderson and Hayes, 2017, 2018), scene description and action description (Henderson et al., 2018; Rehrig et al., 2020), and visual search (Hayes and Henderson, 2019b). These results have also been obtained using viewing tasks that do not require semantic analysis of the scene, such as counting bright, physically salient scene regions (Peacock et al., 2019a), visual search for arbitrarily placed letter targets (Hayes and Henderson, 2019b), and free viewing (Peacock et al., 2019b).

A concern with past meaning mapping work is that viewing patterns tend to show *central fixation bias*, a tendency for viewers to concentrate fixations on the center of a picture (Parkhurst et al., 2002; Tatler, 2007; Tseng et al., 2009; Bindemann, 2010; Rothkegal et al., 2017; van Renswoude et al., 2019). Central fixation bias can be problematic when comparing meaning and image salience if one property is more concentrated in the center of the scene. Studies have shown that image features tend to be more correlated with scene centers due to factors such as photographer bias (van Renswoude et al., 2019) and it is often suggested that there is more meaning in scene centers independent of saliency that could lead to a greater spurious influence of meaning on attention overall (but see: Tatler (2007) who showed that strategy and simple orienting response contribute to center bias independent of photographer bias and image features). Indeed, attempts have been made to disassociate center bias and image content by modifying meaning and saliency maps or removing central fixations *post hoc*. For instance, Hayes and Henderson (2019a) compared the center bias extracted from saliency models to their corresponding full models and found that center bias alone better explained fixation density than the full saliency models, whereas meaning continued to explain fixation density more than center bias alone. In another study, Henderson and Hayes (2017) excluded all central fixations from analyses and found that meaning was more correlated with fixation density than image salience. Finally, Peacock et al. (2019a) used meaning and saliency maps both containing center bias and without center bias and found the advantage of meaning over saliency regardless of center bias. Although these studies provided evidence that meaning predicts eye movements beyond scene centers, they were *post hoc* and correlational in nature and thus were unable to causally dissociate meaning and central fixation bias. Furthermore, these studies changed the predictions of meaning and saliency maps to better account for central fixation bias rather than controlling eye movements themselves. Ideally, we would prevent central fixation bias from happening in the first place in order to test its influence on the meaning advantage more directly.

The goal of the present study, then, was to use an *a priori* manipulation designed to reduce or eliminate the central fixation bias from viewing patterns rather than changing the predictions of meaning and saliency maps. To do so, we adopted a method introduced by Rothkegal et al. (2017). This method involves two changes to common practice: (1) moving the initial fixation location from the center to a quasi-random location in the periphery of the scene, and (2) separating scene onset from the initiation of eye movements using a delayed “go” signal. To test whether our manipulation changed central fixation bias (and thus eye movements to meaning) relative to previous meaning mapping studies, we compared the current data to a previously published study that was identical except that it used central pretrial fixations (Peacock et al., 2019b). If scene centers favor meaning over image salience, then the central pretrial fixation used in Peacock et al. (2019b) could artifactually inflate the apparent relationship between meaning and attention. To test this hypothesis, the current study investigated whether meaning continues to outperform image salience when attention begins in the scene periphery rather than the center.

In summary, the current study sought to compare the relationships of meaning and image salience with eye movements under conditions in which central fixation bias is behaviorally controlled. To accomplish this goal, the initial fixation location was placed in the periphery of the scene and the viewing start time was delayed. We compared attention maps generated by viewers in this peripheral start free viewing task to saliency maps and meaning maps.

## Materials and Methods

### Eyetracking

#### Participants

The sample size was set with an *a priori* stopping rule of 30 participants based on prior experiments using these methods (Peacock et al., 2019a, b). To reach 30 participants, 32 University of California, Davis, undergraduate students with normal or corrected-to-normal vision initially participated in the experiment (27 females, average age = 21.25). All participants were naïve to the purpose of the study and provided verbal consent. The eye movement data from each participant were automatically inspected for artifacts due to blinks or loss of calibration. Following Henderson and Hayes (2017), we averaged the percent signal [(number of good samples/total number of samples) × 100] for each trial and participant using custom MATLAB code. The percent signal for each trial was then averaged for each participant and compared to an *a priori* 75% criterion for signal. Overall, two participants were excluded based on this criterion due to poor eyetracking quality resulting in a total of 30 participants/datasets analyzed. Individual trials that had less than 75% signal were also excluded. In total, no individual trials were excluded based on these criteria.

#### Apparatus

Eye movements were recorded using an EyeLink 1000+ tower mount eyetracker (spatial resolution 0.01°rms) sampling at 1000 Hz (SR Research, 2010b). Participants sat 85 cm away from a 21 inch computer monitor, so that scenes subtended approximately 26.5°× 20° of visual angle at 1024 × 768 pixels. Head movements were minimized by using a chin and forehead rest. Although viewing was binocular, eye movements were recorded from the right eye. The experiment was controlled with SR Research Experiment Builder software (SR Research, 2010a). Fixations and saccades were segmented with EyeLink’s standard algorithm using velocity and acceleration thresholds (30°/s and 9500°/s^2^; SR Research, 2010b). Eye movement data were imported offline into Matlab using the EDFConverter tool. The first fixation was eliminated from analysis because it was experimenter-defined (as opposed to participant-defined). Additionally, fixations that landed off the screen, and any fixations that were less than 50 ms and greater than 1500 ms were eliminated as outliers. Occasionally, saccade amplitudes are not segmented correctly by EyeLink’s standard algorithm, resulting in large values. Given this, saccade amplitudes >25° were also excluded. Fixations corresponding to these saccades were included as long as they met the other exclusion criteria. This outlier removal process resulted in loss of 6.05% of the data across all subjects.

#### Stimuli

Twenty digitized photographs (1024 × 768 pixels) of indoor and outdoor real-world scenes were used as stimuli. Scenes were luminance matched across the scene set by transforming the RGB image of the scene to LAB space and scaling the luminance channel from 0 to 1. Luminance matching was conducted to make sure that there were no overly bright or dark scenes in the experiment and does not change the relative ranking of image salience within a scene. All instruction, calibration, and response screens were luminance matched to the average luminance (*M* = 0.45) of the scenes.

#### Procedure

**FIGURE 2:**
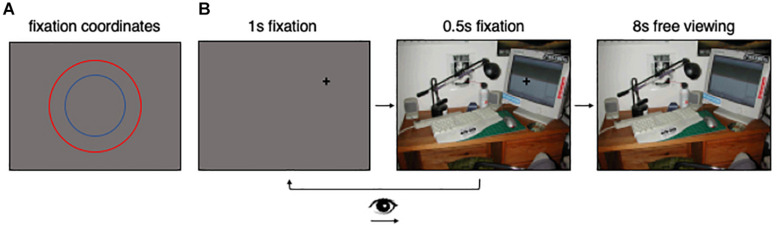
Task figure. **(A)** Shows the locations of the concentric circles that the pretrial fixation coordinates were randomly selected from in this study. **(B)** Is a visual representation of the task.

Participants first completed two practice trials to familiarize them with the task. Prior to the scene viewing portion of the task, participants were instructed to fixate on a black fixation cross (i.e., within a 100 × 100 pixel square window surrounding the cross) on a gray background for 1 s (Figure 2B). The location of the black cross was chosen randomly from the *x,y* coordinate pairs forming two concentric circles centered on the screen (Figure 2A). The concentric circles had radii of 192 and 288 pixels, respectively. During analyses, the eye movements corresponding to the concentric circles (Figure 2A) were collapsed, as the concentric circles provided a method to reduce center bias (via sampling locations across the scene) but we had no theoretical motivation to analyze the data corresponding to the circles separately. After the 1 s period ended, the gray background was replaced with the scene that participants would explore during the scene viewing portion of the experiment (Figure 2B). During this period of time, participants were instructed to maintain gaze on the fixation cross for another 0.5 s. If participants moved their eyes away from the fixation cross during this 0.5 s period, the scene immediately was replaced with a gray screen and participants returned to the beginning of the trial for the same scene (Figure 2B). If fixation was maintained during the 0.5 s period, the cross disappeared, and participants were able to freely move their eyes around the scene for 8 s (Figure 2B). During the scene viewing portion of the experiment, participants were instructed to view each scene naturally, as they would in their daily lives. Given the free viewing nature of the task, participants were not required to provide any responses.

After the practice trials, a 13-point calibration procedure was performed to map eye position to screen coordinates. Successful calibration required an average error of less than 0.49° and a maximum error of less than 0.99°. Presentation of each scene was preceded by a calibration check, and the eye-tracker was recalibrated when the calibration was not accurate.

Each participant viewed all 20 scene stimuli during the task. Scenes were presented in a randomized order for each participant.

### Map Generation

#### Meaning Maps

A subset of the meaning maps generated by Henderson and Hayes (2017) were used in the present study. To create meaning maps, scene-patch ratings were performed by 84 participants on Amazon Mechanical Turk. Participants were recruited from the United States, had a hit approval rate of 99% and 500 hits approved, and were permitted to participate only once. Participants were paid $0.50 per assignment, and all participants provided informed consent. Rating stimuli consisted of the same 20 photographs of real-world scenes used in the eyetracking portion of the experiment. Each scene was decomposed into partly overlapping circular patches at a fine and course spatial scale. The full patch stimulus set consisted of 6,000 fine patches (87-pixel diameter) and 2,160 coarse patches (205-pixel diameter), for a total of 8,160 patches. The ideal meaning-map grid density for each patch size was previously estimated by simulating the recovery of known image properties (i.e., luminance, edge density, and entropy; see Henderson and Hayes, 2018).

Participants were instructed to rate the meaningfulness of each patch based on how informative or recognizable it was on a 6-point Likert scale (very low, low, somewhat low, somewhat high, high, and very high). Prior to the rating task, participants were provided with examples of two low-meaning and two high-meaning scene patches to make sure they understood the rating task. Patches were presented in random order and without scene context, so ratings were based on context-free judgments. Each participant rated 300 random patches. Each unique patch was rated three times by three independent raters for a total of 19,480 ratings across the scene set. Due to the high degree of overlap across patches, each fine patch contained rating information from 27 independent raters and each coarse patch contained rating information from 63 independent raters. Meaning maps were generated by averaging, smoothing, and combining fine and coarse maps from the corresponding patch ratings. The ratings for each pixel at each scale in each scene were averaged, producing an average fine and coarse rating map for each scene. The average rating maps were then smoothed using thin-plate spline interpolation (i.e., thinplateinterp method in MATLAB; MathWorks, Natick, MA, United States). To generate the final meaning map for each scene, the smoothed fine and coarse maps were combined using the simple average (coarse map + fine map / 2).

**FIGURE 3:**
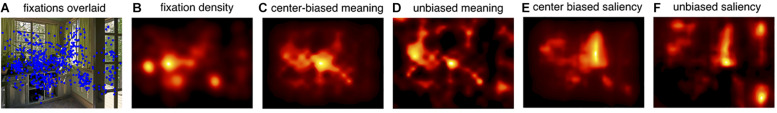
Map examples. **(A)** Shows the example scenes with fixations overlaid and **(B)** is the fixation density map for the example scene. **(C)** Shows the center-biased meaning map and **(D)** shows the unbiased meaning map for the example scene. **(E)** Shows the center-biased saliency map and **(F)** shows the unbiased saliency map for the example scene.

Saliency models typically contain center bias, including the Graph-based Visual Saliency (GBVS) model which is intrinsically center-biased (graph-based differences in computation produces the center bias in GBVS) (Harel et al., 2006). Since meaning maps are not intrinsically center-biased in the same way as GBVS (as meaning maps are based on ratings of isolated scene patches), we added the GBVS center bias to meaning maps to equally weight the centers of meaning and saliency maps. To generate meaning maps containing center-bias, a multiplicative center bias operation was applied to the meaning maps using the center bias present in the GBVS saliency maps. Here, we inverted the “invCenterBias.mat” (i.e., inverted the inverse) included in the GBVS package as an estimate of center bias. From here, we multiplied the resulting center bias and the raw meaning maps to create meaning maps with center bias (Henderson and Hayes, 2017, 2018; Peacock et al., 2019a, b). Note that because meaning maps do not contain intrinsic center bias like GBVS, we used both the original meaning maps containing no center bias and the meaning maps with the center-bias operation applied (Figure 3).

#### Image Salience Maps

Saliency maps for each scene were generated using the GBVS toolbox with default settings (Harel et al., 2006). GBVS is a prominent saliency model that combines maps of low-level image features to create saliency maps (Figure 3). Center bias is a natural feature of GBVS saliency maps. To compare them to the original, unbiased meaning maps, we also generated GBVS maps without center bias (Figure 3). Unbiased GBVS maps were generated using the whitening method (Rahman and Bruce, 2015), a two-step normalization in which each saliency map is normalized to have 0 mean and unit variance. Subsequently, a second, pixel-wise normalization is performed so that each pixel across all the saliency maps has 0 mean and unit variance.

#### Fixation Density Maps

To generate fixation density maps, a fixation frequency matrix based on the locations (*x,y* coordinates) of all fixations (collapsed across both of the concentric circles used to generate pretrial fixation coordinates) was generated across participants for each scene. Then, a Gaussian low-pass filter (from the MIT Saliency Benchmark toolbox)^1^ with a circular boundary and a cutoff frequency of −6 dB (a window size of ∼2° of visual angle) was applied to each matrix to account for foveal acuity and eyetracker error.

#### Histogram Matching

In order to normalize meaning and saliency maps to a common scale, image histogram matching was used with the fixation density map for each scene serving as the reference image for the corresponding meaning and saliency maps for the same scene (Henderson and Hayes, 2017). Image histogram matching is desirable because it normalizes an input image to a reference image, ensuring that the distribution of “power” in the two images is similar. Using the ground-truth fixation density maps as the reference for both meaning and saliency allowed us to directly compare the meaning and saliency maps. The “imhistmatch” function from the Matlab Image Processing Toolbox was used to accomplish image histogram matching.

## Results

### Center Bias

To assess whether the tendency to fixate scene centers was reduced by employing peripherally located fixation crosses with delayed eye movements (Rothkegal et al., 2017), we tested the strength of the central fixation bias in both a representative meaning mapping study that contained central fixation bias and employed a central pretrial fixation (Peacock et al., 2019b) and the current peripheral start experiment. Central start refers to the Peacock et al. (2019b) and peripheral start refers to the current study.

**FIGURE 4:**
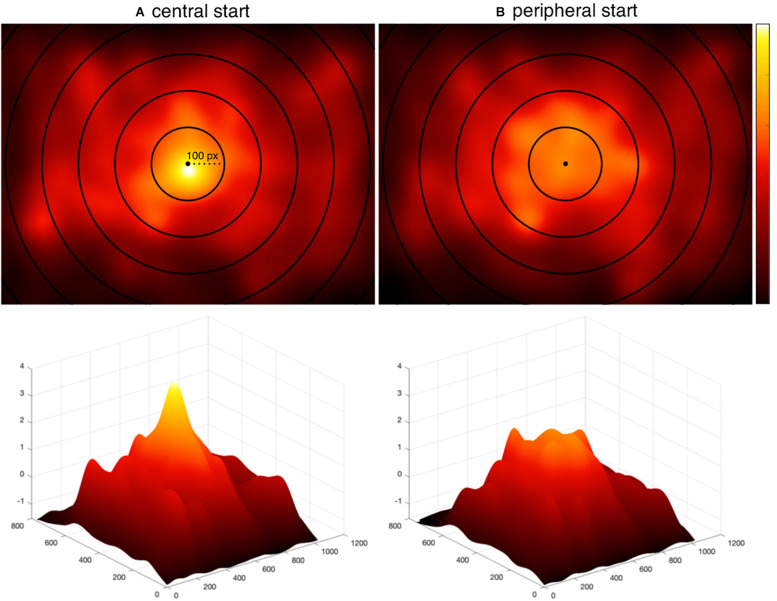
Fixation distributions. The distribution of all fixations aggregated across participants and scenes **(A)** from Peacock et al. (2019b) using a centrally located fixation cross, and **(B)** from the current experiment using a peripherally located fixation cross with delayed trial start. Concentric circles are overlaid on each map to show the extent of central bias. The most inner circle has a radius of 100 pixels and each circle increments the radius by 100 pixels. The second row visualizes the same heat maps in three dimensions. Heat maps are z-normalized to a common scale with black representing no fixations and white representing the highest density of fixations.

To test the strength of the center bias reduction in the current study, we generated fixation density maps for each scene in each study and then z-normalized the fixation density maps for each scene to one another. Because the largest difference in center bias was observed within a 200-pixel window around center (Figure 4), we focused an initial center bias analysis on these pixels. After excluding regions of each map that were not contained within this window, the values at each pixel of each map were then converted to a vector and subtracted from one another (i.e., central start pixels – peripheral start pixels) to calculate a difference score of center bias for each scene. An average difference score for each scene was calculated by averaging the difference scores for each pixel. A positive difference score indicated there was greater center bias in the central start study for that scene and a negative difference score indicated there was greater center bias in the current, peripheral-start study for that scene.

A two-tailed one-sample *t*-test showed that center bias was significantly reduced in the current peripheral start study relative to the central start study (*M* = 0.28, *SD* = 0.42): *t*(19) = 3.05, *p* = 0.006, 95% CI = [0.09, 0.48]. The degrees of freedom refer to the total number of scenes minus one (N–1) and confidence interval indicates the range of values that were 95% certain to include the true population mean. To test how the manipulation influenced center bias across the span of scenes, we also conducted the same analysis using all of the pixels. Here, the result replicated (*M* = 0.04, *SD* = 0.03): *t*(19) = 5.17, *p* < 0.001, 95% CI = [0.02, 0.06]. We further visualize this in Figure 4 with heat maps representing all fixations across all participants and scenes in the present study and the Peacock et al. (2019b) central start study. Both the analysis and plots show that the strong central bias in the central start experiment (Peacock et al., 2019b) was reduced with the peripheral start paradigm used in the current study.

### Eye Movements

#### Whole Scene Analyses

Given that the current study successfully reduced the central fixation bias, we next sought to understand the relationship between attention to meaningful and salient scene regions. Linear Pearson correlations (Bylinskii et al., 2015) were used to quantify how much variance in fixation densities meaning and saliency accounted for. The CC.m function from the MIT saliency benchmark toolbox^2^ was used to calculate the Pearson correlation. We chose CC.m because it has been used to evaluate the various metrics included in the MIT saliency benchmark (Bylinskii et al., 2015). The function works by first normalizing the to-be-correlated maps. It then converts the two-dimensional map arrays to one-dimensional vectors and correlates these vectors. The output of the function is then squared to calculate the shared variance explained by meaning and saliency. Two-tailed, paired *t*-tests were used to test the relative ability of the meaning and saliency maps to predict the variance in fixation density. We note that because statistics are performed on the scene-level and not the participant-level, the degrees of freedom in the following analyses refer to the number of scenes used in the experiment minus one.

To investigate how meaning and salience independently accounted for the variance in fixation densities, semi-partial correlations were used. Semi-partial correlations capture the amount of total variance in fixation densities that can be accounted for with the residuals from meaning or saliency after removing the intercorrelation between meaning and saliency. In other words, semi-partial correlations show the total variance in fixation densities that can be accounted for by the meaning-independent variance in salience and the salience-independent variance in meaning. Two-tailed one-sample *t*-tests were employed to test whether the unique variance in attention explained by each map type was significantly greater than zero.

In past meaning mapping studies including Peacock et al. (2019b), center-biased meaning and saliency maps were used to predict eye movements, as there was significant central fixation bias during viewing 2019b. In the present study, we therefore first used center-biased prediction maps to more equally compare the original free viewing results to those of the current study and because GBVS maps are intrinsically center-biased. Because meaning maps do not contain this intrinsic center bias, however, we also conducted analyses with unbiased meaning and saliency maps. If the advantage of meaning over image salience in previous meaning mapping studies using the central start position, such as in Peacock et al. (2019b), was a function of center bias, then that advantage should be reduced in the present study. On the other hand, if the advantage of meaning over image salience is a general phenomenon and not a function of center bias, then we should continue to see that advantage.

**FIGURE 5:**
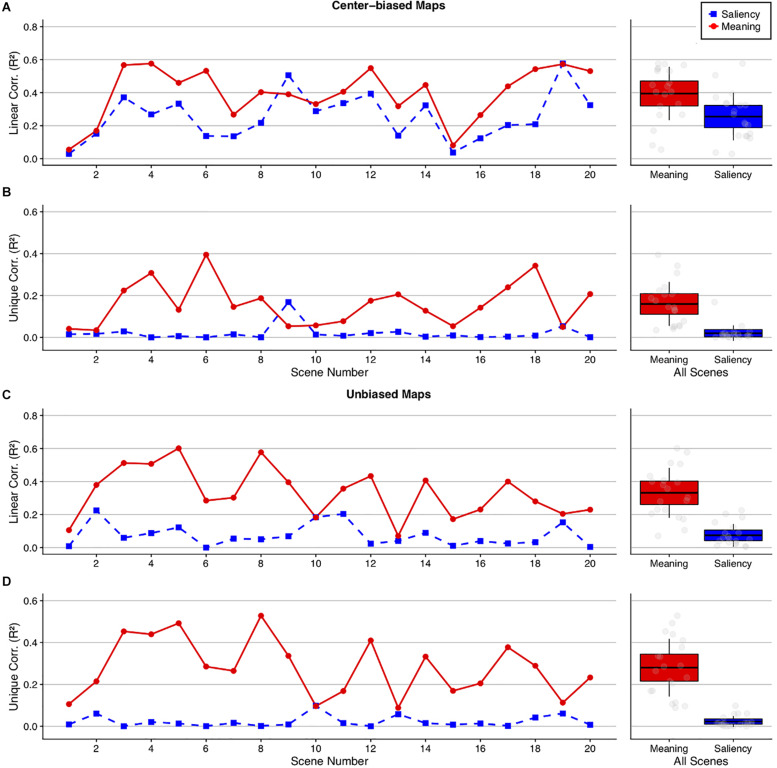
Squared linear and semi-partial correlations by scene comparing meaning and image salience. Line plots show the **(A,C)** squared linear and **(B,D)** semi-partial correlations between the fixation density maps, meaning (red circles), and image salience (blue squares) using **(A,B)** center-biased and **(C,D)** unbiased prediction maps. The scatter plots show the grand mean (black horizontal line), 95% confidence intervals (colored boxes), and 1 standard deviation (black vertical line), for meaning and image salience across all 20 scenes for each analysis.

Using center-biased meaning and saliency maps (Figure 5), meaning explained 40% (*M* = 0.40, *SD* = 0.16) and image salience explained 26% of the variance in fixation density (*M* = 0.26, *SD* = 0.15) with linear correlations, *t*(19) = 5.07, *p* < 0.001, 95% CI = [0.08, 0.20] (Figure 5). For the semi-partial correlations, meaning explained 16% (*M* = 0.16, *SD* = 0.11) (*t*(19) = 6.79, *p* < 0.001, 95% CI = [0.11, 0.21]) and saliency explained 2% of the variance in fixation density (*M* = 0.02, *SD* = 0.04) (*t*(19) = 2.40, *p* = 0.03, 95% CI = [0.003, 0.04]). Although meaning and image salience explained significant overall variance in fixation density, salience predicted very little unique variance.

Using unbiased meaning and saliency maps (Figure 5), meaning explained 33% (*M* = 0.33, *SD* = 0.15) whereas image salience explained 7% of the variance in fixation density (*M* = 0.07, *SD* = 0.07) with linear correlations, *t*(19) = 7.44, *p* < 0.001, 95% CI = [0.19, 0.33]. For the semi-partial correlations, meaning explained a unique 28% (*M* = 0.28, *SD* = 0.14) (*t*(19) = 9.09, *p* < 0.001, 95% CI = [0.22, 0.35]) whereas saliency explained only a unique 2% of the variance (*M* = 0.02, *SD* = 0.03) (*t*(19) = 3.74, *p* = 0.001, 95% CI = [0.01, 0.04]). As with the center biased maps, meaning and saliency explained significant overall variance in fixation density but meaning predicted substantial variance whereas saliency did not.

Finally, the strongest test of whether meaning was superior in predicting eye movements relative to image salience despite central fixation bias was to compare the unbiased meaning maps, which are not upweighted at scene centers where fixations tend to land, to center-biased saliency maps. To test this, the unbiased meaning linear correlations and the center-biased salience linear correlations were submitted to a paired *t*-test. The results showed that the unbiased meaning maps predicted fixation densities significantly better (33%) than the center-biased saliency maps (26%): *t*(19) = 2.05, *p* = 0.05, 95% CI = [−0.001, 0.15]. Unbiased meaning explained 17% unique variance (*M* = 0.17, *SD* = 0.09; *t*(19) = 8.38, *p* < 0.001, 95% CI = [0.13, 0.22]) and center-biased saliency explained only 10% of this variance (*M* = 0.10, *SD* = 0.09; *t*(19) = 4.82, *p* < 0.001, 95% CI = [0.06, 0.14]), suggesting that even when meaning maps are not upweighted in scene centers, they can outperform saliency maps that do contain center bias.

As shown in Table 1, the overall magnitudes of values and effects were very similar between the present peripheral start experiment and our previous central start experiment.

**TABLE 1:** Comparison between central start and peripheral start experiments using the meaning and saliency maps to predict the overall pattern of attention.

**Center-biased maps**		
**Correlation type**	**Central start**	**Peripheral start**
Linear Meaning	*M* = 0.39, *SD* = 0.14	*M* = 0.40, *SD* = 0.16
Linear Image Salience	*M* = 0.24, *SD* = 0.14	*M* = 0.26, *SD* = 0.15
Paired *t*-test	*t*(19) = 7.08, *p* < 0.001, 95% CI = [0.10, 0.19]	*t*(19) = 5.07, *p* < 0.001, 95% CI = [0.08, 0.20]
Unique Meaning	*M* = 0.16, *SD* = 0.07	*M* = 0.16, *SD* = 0.11
One-sample *t*-test	*t*(19) = 9.52, *p* < 0.001, 95% CI = [0.13, 0.20]	*t*(19) = 6.79, *p* < 0.001, 95% CI = [0.11, 0.21]
Unique Image Salience	*M* = 0.02, *SD* = 0.03	*M* = 0.02, *SD* = 0.04
One-sample *t*-test	*t*(19) = 2.37, *p* = 0.03, 95% CI = [0.002, 0.03]	*t*(19) = 2.40, *p* = 0.03, 95% CI = [0.003, 0.04]

**Unbiased maps**		
Linear Meaning	*M* = 0.33, *SD* = 0.12	*M* = 0.33, *SD* = 0.15
Linear Image Salience	*M* = 0.08, *SD* = 0.08	*M* = 0.07, *SD* = 0.07
Paired *t*-test	*t*(19) = 8.07, *p* < 0.001, 95% CI = [0.18, 0.31]	*t*(19) = 7.44, *p* < 0.001, 95% CI = [0.19, 0.33]
Unique Meaning	*M* = 0.27, *SD* = 0.11	*M* = 0.28, *SD* = 0.14
One-sample *t*-test	*t*(19) = 10.73, *p* < 0.001, 95% CI = [0.22, 0.33]	*t*(19) = 9.09, *p* < 0.001, 95% CI = [0.22, 0.35]
Unique Image Salience	*M* = 0.03, *SD* = 0.04	*M* = 0.02, *SD* = 0.03
One-sample *t*-test	*t*(19) = 3.32, *p* = 0.004, 95% CI = [0.01, 0.05]	*t*(19) = 3.74, *p* = 0.001, 95% CI = [0.01, 0.04]

*Comparisons include center bias and unbiased meaning and saliency maps, and linear and semi partial correlations. The central start data are from Peacock et al. (2019b).*

#### Early Fixation Analyses

It has been hypothesized that early fixations may be more directly controlled by image salience than subsequent fixations (Parkhurst et al., 2002; Borji et al., 2013; Anderson et al., 2015). Although data from our prior work has not supported that hypothesis (Henderson and Hayes, 2017, 2018; Henderson et al., 2018; Peacock et al., 2019a, b), these studies used a central fixation position, which arguably could have favored meaning over salience. Since central fixation bias was significantly reduced in the current study compared to our central start study (Figure 4), we conducted an additional analysis focused specifically on early fixations to test whether meaning continues to account for significantly greater variance in fixation density compared to image salience. The data were submitted to an ordinal fixation analysis for the first three subject-generated fixations, in which fixation density maps were produced for each sequential fixation in each scene (Henderson and Hayes, 2017, 2018; Henderson et al., 2018; Peacock et al., 2019a, b). For each fixation, analyses proceeded as in the whole scene analyses, and *p*-values were corrected for multiple comparisons using the Bonferroni correction. If greater early attention to meaning versus salience observed in our previous studies was a function of center bias, then that advantage should be eliminated here. If greater early attention to meaning generalizes beyond center bias, as our previous statistical control of center bias suggests (Henderson and Hayes, 2017; Hayes and Henderson, 2019a; Peacock et al., 2019a), then the results should continue to show an advantage of meaning over salience here even though center bias was reduced.

**FIGURE 6:**
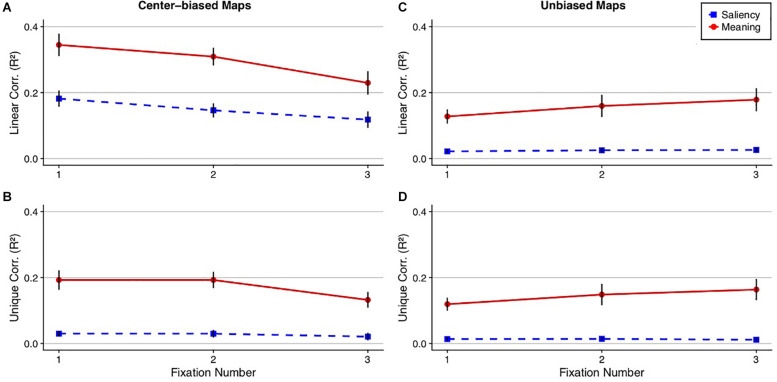
Ordinal fixation analysis comparing meaning and image salience. The line plots show **(A,C)** the squared linear and **(B,D)** semi-partial correlations between the fixation density maps, meaning (red circle), and image salience (blue square) as a function of fixation number collapsed across scenes using the **(A,B)** center-biased and **(C,D)** unbiased prediction maps. Error bars represent the standard error of the mean.

For the center-biased maps, meaning accounted for 35, 31, and 23% and saliency accounted for 18, 15, and 12% of the variance in the first three fixations, respectively, for the linear correlations (Figure 6), with all three fixations showing a significant meaning advantage over image salience in predicting fixation density (fixation 1: *t*(19) = 4.83, Bonferroni-corrected *p* < 0.001, 95% CI = [0.09, 0.23]; fixation 2: *t*(19) = 5.37, Bonferroni-corrected *p* < 0.001, 95% CI = [0.10, 0.23]; fixation 3: *t*(19) = 4.03, Bonferroni-corrected *p* < 0.001, 95% CI = [−0.05, 0.17]). For the semi-partial correlations, meaning accounted for a significant 19, 19, and 13% of the unique variance in the first three fixations (fixation 1: *t*(19) = 6.53, Bonferroni-corrected *p* < 0.01, 95% CI = [0.13, 0.25]; fixation 2: *t*(19) = 7.81, Bonferroni-corrected *p* < 0.001, 95% CI = [0.14, 0.24]; fixation 3: *t*(19) = 5.56, Bonferroni-corrected *p* < 0.001, 95% CI = [0.08, 0.18]) and saliency accounted for 3, 3, and 2% of the unique variance in the first three fixations, respectively. Saliency only explained a significant amount of the unique variance on fixation 1 but not fixations 2 or 3 (fixation 1: *t*(19) = 3.69, Bonferroni-corrected *p* = 0.01, 95% CI = [0.01, 0.05]; fixation 2: *t*(19) = 2.60, Bonferroni-corrected *p* = 0.11, 95% CI = [0.006, 0.05]; fixation 3: *t*(19) = 1.80, Bonferroni-corrected *p* = 0.52, 95% CI = [−0.003, 0.05]) In total, this suggests that meaning was a significantly better predictor than saliency when considering the earliest of eye movements.

For the unbiased maps, meaning accounted for 13, 16, and 18% and saliency accounted for 2, 3, and 3% of the variance in the first three fixations for the linear correlations (Figure 6), with significant differences between meaning and salience for all three fixations (fixation 1: *t*(19) = 4.68, Bonferroni-corrected *p* = 0.001, 95% CI = [0.06, 0.15]; fixation 2: *t*(19) = 3.92, Bonferroni-corrected *p* = 0.003, 95% CI = [0.06, 0.21]; fixation 3: *t*(19) = 4.49, Bonferroni-corrected *p* = 0.001, 95% CI = [0.08, 0.22]). The results did not change for the semi-partial correlations, with meaning accounting for a significant 12, 15, and 16% of the variance in the first three fixations (fixation 1: *t*(19) = 6.10, Bonferroni-corrected *p* < 0.001, 95% CI = [0.08, 0.16]; fixation 2: *t*(19) = 4.60, Bonferroni-corrected *p* = 0.001, 95% CI = [0.08, 0.22]; fixation 3: *t*(19) = 5.10, Bonferroni-corrected *p* < 0.001, 95% CI = [0.10, 0.23]) whereas saliency accounted for a non-significant 2, 3, and 3% of the variance in the first three fixations (fixation 1: *t*(19) = 2.43, Bonferroni-corrected *p* = 0.15, 95% CI = [0.002, 0.03]; fixation 2: *t*(19) = 2.96, Bonferroni-corrected *p* = 0.05, 95% CI = [0.004, 0.03]; fixation 3: *t*(19) = 2.85, Bonferroni-corrected *p* = 0.06, 95% CI = [0.003, 0.02]). The results considering the unbiased maps replicated the center biased maps in that meaning predicted significantly greater variance in fixation density than image salience. Furthermore, salience predicted no unique variance in attention when meaning was partialed out but when saliency was partialed out, meaning continued to account for unique variance in attention.

To test whether unbiased meaning maps were superior in predicting eye movements relative to center-biased image salience maps on a fixation by fixation basis, the unbiased meaning linear correlations and the center-biased salience linear correlations for each fixation were submitted to paired *t*-tests corrected for multiple comparisons via the Bonferroni correction. The results showed that for the first fixation, center-biased saliency had a numerical but not a significant advantage over unbiased meaning: *t*(19) = −2.22, Bonferroni-corrected *p* = 0.12, 95% CI = [−0.11, −0.003]. For the second and third fixations, meaning had a numerical, non-significant advantage over image salience (fixation 2: *t*(19) = 0.40, Bonferroni-corrected *p* = 1.00, 95% CI = [−0.06, 0.09]; fixation 3: *t*(19) = 1.87, Bonferroni-corrected *p* = 0.23, 95% CI = [−0.007, 0.13]). Unbiased meaning explained significant unique variance in the first three fixations (Fixation 1: *M* = 0.06, *SD* = 0.04; *t*(19) = 6.12, Bonferroni-corrected *p* < 0.001, 95% CI = [0.04, 0.08]; Fixation 2: *M* = 0.10, *SD* = 0.11; *t*(19) = 4.01, Bonferroni-corrected *p* = 0.005, 95% CI = [0.05, 0.15]; Fixation 3: *M* = 0.11, *SD* = 0.10; *t*(19) = 4.99, Bonferroni-corrected *p* < 0.001, 95% CI = [0.07, 0.16]) and image salience explained unique variance in the first two fixations (Fixation 1: *M* = 0.11, *SD* = 0.08; *t*(19) = 6.04, Bonferroni-corrected *p* < 0.001, 95% CI = [0.07, 0.15]; Fixation 2: *M* = 0.08, *SD* = 0.07; *t*(19) = 5.39, Bonferroni-corrected *p* < 0.001, 95% CI = [0.05, 0.11]) but not the third fixation (*M* = 0.06, *SD* = 0.09; *t*(19) = 2.87, Bonferroni-corrected *p* = 0.06, 95% CI = [0.02, 0.10]).

Although only 10.70% (SD = 0.13) of trials were repeated due to participants failing to maintain fixation during scene onset, we reran the analyses excluding these trials and found the results to be unchanged. This suggests that multiple previews of scenes did not drive any of the reported effects.

As shown in Table 2, the earliest fixations showed similar effects of meaning over saliency in the present study as the earlier central start experiment (Peacock et al., 2019b), contrary to the hypothesis that the early fixation advantage of meaning over image salience previously observed was simply due to center bias from the initial fixation locations used in previous meaning mapping studies.

**TABLE 2:** Comparison between peripheral start (current study) and central start (Peacock et al., 2019b) experiments using meaning (percentage of variance explained) and saliency (percentage of variance explained) to predict early fixations.

**Center-biased maps**						
	**Central start**			**Peripheral start**		
**Correlation type**	**Fix 1**	**Fix 2**	**Fix 3**	**Fix 1**	**Fix 2**	**Fix 3**
Linear meaning	38%	31%	20%	35%	31%	23%
Linear image salience	10%	15%	11%	18%	15%	12%
Meaning advantage?	Yes	Yes	Yes	Yes	Yes	Yes
Unique meaning	30%	19%	12%	19%	19%	19%
Significant?	Yes	Yes	Yes	Yes	Yes	Yes
Unique image salience	2%	3%	3%	3%	3%	2%
Significant?	Yes	Yes	Yes	Yes	No	No

**Unbiased maps**						
Linear meaning	8%	15%	15%	13%	16%	18%
Linear image salience	2%	4%	4%	2%	3%	3%
Meaning advantage?	Yes	Yes	Yes	Yes	Yes	Yes
Unique meaning	7%	13%	14%	12%	15%	16%
Significant?	Yes	Yes	Yes	Yes	Yes	Yes
Unique image salience	1%	2%	2%	2%	3%	3%
Significant?	No	No	No	No	No	No

Overall, the results are consistent with previous meaning mapping work using a traditional central fixation start location (Henderson and Hayes, 2017, 2018; Henderson et al., 2018; Peacock et al., 2019a, b; Rehrig et al., 2020) in which we found that early eye movements were more related to meaning than saliency. The present findings verify that the advantage of meaning over salience observed by previous meaning mapping studies was not simply due to an advantage for meaning at scene centers induced by the use of an initial central fixation location. Furthermore, this conclusion is strengthened when only the earliest fixations are analyzed. Overall, these findings show that when employing a paradigm that reduces central fixation bias, early fixations are still better explained by meaning than by image salience.

#### Scene-Dependent and Independent Spatial Biases in Meaning and Saliency Maps

As patches of meaning and salient locations are differently distributed across the images, it is theoretically possible that fixations are not predicted or explained by meaning or salience but that rather a third factor that drives the spatial distributions of meaning, image salience, and fixations. Center bias is one such factor. If meaning/saliency maps are capturing scene-specific distributions of meaning/saliency (as opposed to scene-independent spatial biases in eye movements, such as center bias), then a meaning/saliency map for a given scene should be significantly more related to fixation densities from the same scene than to fixation densities from another scene. However, if meaning and saliency maps are simply capturing center bias (scene-independent spatial biases in viewing), then the meaning and saliency map for a given scene should not be any more related to fixation densities from the same scene or another.

**FIGURE 7:**
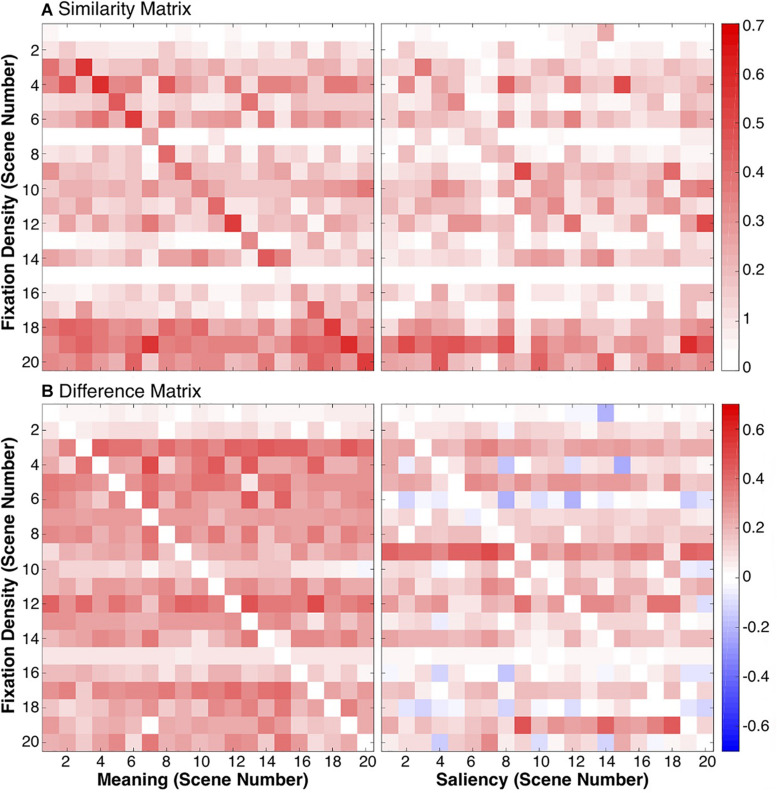
Similarities between meaning/saliency maps and fixation densities. The similarity matrices **(A)** show the squared linear correlations between fixation densities and meaning/image salience maps for each scene combination. The difference matrices **(B)** show the difference between the correlations of fixation densities and meaning/saliency for the same scene and correlations of fixation densities and meaning/saliency from different scenes.

To test this, we calculated a scene-by-scene fixation density squared linear correlation to the meaning and saliency maps. Because there were 20 scenes, this produced two 20×20 similarity matrices, one for meaning and one for saliency (Figure 7A). If each model is capturing scene-dependent variance, then the diagonal of the similarity matrix should have a larger value than the off-diagonal value. Conversely, if the models are only capturing spatial bias, then the matrices should be uniform.

Difference calculations were computed for both models, again producing two 20×20 difference matrices, one for meaning and one for saliency (Figure 7B). Difference scores were computed by taking the difference between each model correlated with fixation densities from the same scenes (i.e., the diagonals from Figure 7A) and the correlations computed between the same meaning/saliency maps and the fixation densities from all the other scenes (off-diagonals in Figure 7A). If a given meaning map or saliency map was more strongly correlated with the fixation densities from the same scene than another scene, then the difference score was positive. If a given meaning or saliency map was more strongly correlated with fixation densities from another scene than the same scene, then the difference score was negative. Difference scores along the diagonal were 0 (Figure 7B).

The average difference score for each scene was then computed and submitted to a one-sample *t*-test comparing the difference scores for meaning (*M* = 0.23, *SD* = 0.02) and saliency (*M* = 0.12, *SD* = 0.03) to 0. Overall, meaning and saliency maps were significantly more related to fixation densities from the same scene than other scenes (meaning: *t*(19) = 51.43, *p* < 0.001, 95% CI = [0.22, 0.24]; saliency: *t*(19) = 16.15, *p* < 0.001, 95% CI = [0.10, 0.13]). In both cases, meaning and saliency predict scene-specific eye movements significantly better than would be expected by chance. However, a paired *t*-test comparing the difference scores showed that meaning maps for a given scene were significantly more related to fixation densities for a given scene than image salience (*t*(19) = 14.98, *p* < 0.001, 95% CI = [0.09, 0.12]), suggesting that meaning captured more scene-specific meaning not related to scene-independent spatial biases in viewing than salience. In both cases, meaning and saliency are predicting scene-specific eye movements significantly better than would be expected by chance.

## General Discussion

Recent work in real-world attentional guidance has shown that meaning maps representing the semantic features of local scene regions are more highly related to fixation distributions than are saliency maps representing image feature differences, a result that has been replicated across a number of viewing tasks (Henderson and Hayes, 2017, 2018; Henderson et al., 2018; Hayes and Henderson, 2019b; Peacock et al., 2019a, b; Rehrig et al., 2020). However, centers of photographs may contain greater meaningful information and image features than in scene peripheries, and for that reason participants might strategically fixate centrally (Parkhurst et al., 2002; Tatler, 2007; Tseng et al., 2009; Bindemann, 2010; Rothkegal et al., 2017; van Renswoude et al., 2019), conflating whether meaning actually guides attention better than image salience or whether this phenomenon is due to central fixation bias. Although previous meaning map studies have made attempts to tackle this issue by modifying meaning and saliency maps or eye movements in a *post hoc* fashion [i.e., removing scene centers (Henderson and Hayes, 2017), directly comparing center bias-only saliency models to full saliency models (Hayes and Henderson, 2019a) or by using center-biased and unbiased meaning and saliency maps to predict fixations (Peacock et al., 2019a)], to date there has been no formal attempt to manipulate the extent to which participants attend to scene centers *a priori* and how such a manipulation interacts with meaning and saliency.

The purpose of the current study was consequently to test whether meaning continues to produce an advantage over saliency when central fixation bias is experimentally reduced. To reduce center bias, we used a recent method in which the location of the pretrial fixation cross is presented peripherally, and the first eye movement is delayed after scene onset (Rothkegal et al., 2017). We then compared our data to Peacock et al. (2019b), an identical meaning mapping study except with an initial central starting fixation.

There were three main results. First, to validate that our peripheral fixation manipulation reduced center bias, we compared the amount of center bias present here against the amount of center bias present in an identical experiment with central fixation (Peacock et al., 2019b). We found that the amount of center bias was significantly reduced here relative to Peacock et al. (2019b), a finding that converges with Rothkegal et al. (2017).

Second, even with central bias reduced, we found that meaning predicted significantly greater variance in fixation density than image salience. When the variance explained by meaning was controlled, image salience alone was unable to account for variance in fixation density, but when the variance explained by image salience was statistically controlled, meaning still accounted for variance in fixation density. An ordinal fixation analysis showed that meaning is more related to the guidance of eye movements than image salience at the earliest fixations, contrary to the proposal that image salience preferentially guides early attention (Henderson and Hollingworth, 1999; Henderson and Ferreira, 2004; Anderson et al., 2015; Anderson and Donk, 2017). These results held true for analyses using both traditional meaning and saliency maps containing center bias as well as maps in which center bias was removed.

We also assessed whether unbiased meaning maps predicted fixation densities better than center-biased saliency maps. The main analysis showed that unbiased meaning predicted eye movements above and beyond center-biased saliency, despite not being upweighted in scene centers. For the ordinal fixation analyses, saliency had a numerical advantage on the first fixation which was likely due to the artifactual upweighting that center-bias generates in early viewing relative to maps not containing center bias (Peacock et al., 2019a, b). However, for the second and third fixations, meaning had a numerical advantage over image salience. This suggests that even when meaning maps are not upweighted in scene centers, they can outperform saliency maps that do contain center bias. In total, the finding that meaning still explained eye movements better than image salience when the tendency to fixate centrally was reduced indicates that the eye movement guidance advantage of meaning over image salience is not an artifact of central fixation bias found in previous meaning mapping work.

A final analysis tested whether the spatial distributions of meaning and image salience are driven by scene-independent spatial biases in viewing (center bias) or whether these maps truly capture scene-specific distributions of meaning and saliency. The results showed that meaning is driven by scene-specific information not related to scene-independent spatial biases in viewing whereas image salience is driven by some scene-specific information but also captures general spatial biases in viewing (i.e., center bias) not tied to the saliency distribution of a specific scene. This result converges with Hayes and Henderson (2019a) who found that when center bias is extracted from a given saliency model, this center bias alone predicts eye movements better than the original saliency model, but that center bias does not predict fixation locations better than meaning. Together, the current result and the finding from Hayes and Henderson (2019a) advocates for a model in which scene centers attract fixations beyond image salience but not beyond meaning.

### Conclusion

The results of the present study were consistent with past meaning mapping work demonstrating that meaning accounts for the spatial distribution of fixations better than image salience during scene viewing, and extended those findings to a task in which central fixation bias was experimentally reduced *a priori*. Findings indicated that meaning distributions are driven by scene-dependent information unrelated to center bias whereas saliency distributions are driven by scene-dependent information and center bias. We conclude that meaning plays the central role in attentional prioritization in scenes with center bias controlled.

## Data Availability Statement

The raw data supporting the conclusions of this article will be made available by the authors, without undue reservation.

## Ethics Statement

The studies involving human participants were reviewed and approved by the University of California, Davis, IRB. Written informed consent for participation was not required for this study in accordance with the National Legislation and the Institutional Requirements.

## Author Contributions

CP and JH conceived and designed the study. CP collected the data, analyzed the data, and wrote the manuscript. CP, TH, and JH conceived of the analyses. All authors contributed to the article and approved the submitted version.

## Conflict of Interest

The authors declare that the research was conducted in the absence of any commercial or financial relationships that could be construed as a potential conflict of interest.

## Footnotes

1 https://github.com/cvzoya/saliency/blob/master/code_forMetrics/antonio Gaussian.m

2 https://github.com/cvzoya/saliency/blob/master/code_forMetrics/CC.m
